# mTOR and MAPK: from localized translation control to epilepsy

**DOI:** 10.1186/s12868-016-0308-1

**Published:** 2016-11-17

**Authors:** Helena F. Pernice, Rico Schieweck, Michael A. Kiebler, Bastian Popper

**Affiliations:** Department of Anatomy and Cell Biology, Biomedical Center (BMC), Medical Faculty, Ludwig-Maximilians-University (LMU), Großhaderner Straße 9, 82152 Planegg-Martinsried, Germany

**Keywords:** RBP, mTOR, MAPK, ERK, Epilepsy

## Abstract

**Background:**

Epilepsy is one of the most common neurological diseases characterized by excessive hyperexcitability of neurons. Molecular mechanisms of epilepsy are diverse and not really understood. All in common is the misregulation of proteins that determine excitability such as potassium and sodium channels as well as GABA receptors; which are all known as biomarkers for epilepsy. Two recently identified key pathways involve the kinases mechanistic target of rapamycin (mTOR) and mitogen-activated protein kinases (MAPK). Interestingly, mRNAs coding for those biomarkers are found to be localized at or near synapses indicating a local misregulation of synthesis and activity.

**Results:**

Research in the last decade indicates that RNA-binding proteins (RBPs) responsible for mRNA localization, stability and translation mediate local expression control. Among others, they are affected by mTOR and MAPK to guide expression of epileptic factors. These results suggest that mTOR/MAPK act on RBPs to regulate the fate of mRNAs, indicating a misregulation of protein expression at synapses in epilepsy.

**Conclusion:**

We propose that mTOR and MAPK regulate RBPs, thereby guiding the local expression of their target-mRNAs encoding for markers of epilepsy. Thus, misregulated mTOR/MAPK-RBP interplay may result in excessive local synthesis of ion channels and receptors thereby leading to hyperexcitability. Continuous stimulation of synapses further activates mTOR/MAPK pathway reinforcing their effect on RBP-mediated expression control establishing the basis for epilepsy. Here, we highlight findings showing the tight interplay between mTOR as well as MAPK with RBPs to control expression for epileptic biomarkers.

## Background

Epilepsy is one of the most common neurological diseases, affecting about 65 million people in the world [1], therein 8.5 per 1000 population in the US [2] and 18 per 1000 population in Europe [3]. The disease affects life quality through discrimination and segregation of patients [4]. Furthermore, therapies have high economic costs of up to $13.8 billion in Europe [5]. Available therapies usually target symptoms and are still ineffective in 30% of all cases [6]. It is commonly believed that abnormal synchronous firing in a hyperexcitable neuronal network results in epileptic seizures [7]. Generally, epilepsy can be differentiated according to the region of its generation: Generalized seizures origin from both hemispheres, whereas focal epilepsy derives from one specific area of the brain, most commonly the temporal lobe or the limbic system, which results in temporal lobe epilepsy (TLE) [8]. Idiopathic epilepsies that can take both generalized and focal forms, have a genetic or epigenetic cause, classifying genetic epilepsy as one specific group [9]. In the last decade, immense effort was made to identify mutations ending up with a list of genetic risk factors causing epilepsy [10, 11] (Table 1). In 40% of the cases, however, the etiology of epilepsy is unknown. This emphasizes the need for a better understanding of genetic causes forming the basis of putative therapies.

**Table 1:** Epilepsy targets that are regulated by RBPs and dependent on mTOR/MAPK activity

	Effect	Pathway	Epilepsy related targets		Link to epilepsy
			Gene	Protein	
FMRP	Translational repression and dendritic RNA transport [48]	mTOR [57–61]	*CaMKIIα*	CaMKIIα [20, 48]	Childhood seizures in patients and mice with FXS [27, 56]
		MAPK [20, 62, 65]	*KCNC1*	Kv3.1 [52]	
			*KCND2*	Kv4.2 [54]	
			*CACNA1B*	Cav2.2 [53]	Audiogenic seizures in rats with FXS [62]
			*KCNMA1*	BK channel [51]	
			*KCNT1*	Slack channel [50]	
HuD	RNA stabilization	mTOR [76, 77, 79]	*CaMKIIα*	CaMKIIα [77]	Increased protein level in rats with kainate induced seizures [70]
	Splicing control	MAPK [76, 80]	*KCNA1*	Kv1.1 [69]	
	Neuronal differentiation and plasticity [67]		*GLS*	Glutaminase [73]	Increased susceptibility to audiogenic seizures in mice [28]
HuR	RNA stabilization	MAPK [82–85]	*GAP-43*	GAP-43 [68, 72]	Pentylenetetrazol-induced seizures in mice [82]
	Splicing control [86]				
	Cellular stress response [67]				
CREB	Translational activator [89]	MAPK [40]	*Bdnf*	BDNF [29, 92]	Epileptic seizures in animal models of epilepsy and human patients [29]

RBPs, their general effect on gene expression, involvement in mTOR and/or MAPK pathways, their mRNA targets, and encoded proteins as well as their link to epilepsy in animal models and human patients are depicted

The genetic risk factors that have been found are diverse and are involved in regulation of various pathways affecting cell death, morphology and neurogenesis. One aspect that is essential for all models of epileptogenesis is the dysregulation of synaptic function. This includes presynaptic vesicle release, postsynaptic receptor expression and ion channel expression, which have provided new approaches on therapy for epilepsy [12, 13].

Recently, it has been shown that the mechanistic target of rapamycin (mTOR) and mitogen-activated protein kinases (MAPK) are important regulators of synaptic excitability involved in cognitive impairment and epilepsy in animal models as well as human disease [14–16]. Those kinases regulate gene expression in neurons in a stimulus-dependent manner [17, 18]. Both pathways can be activated i.e. by long-term potentiation (LTP), a process characterized by enhanced transmission between simultaneously activated synapses. Still, not all neuronal stimulation paradigms lead to the activation of both mTOR and MAPK pathways [19, 20]. Further research is necessary to describe the exact mechanisms and kind of neuronal excitation that leads to hyperactivity. Regardless, multiple key studies show an effect of mTOR and MAPK on general expression control [21, 22]. Remarkably, recent research has shown that both mTOR and MAPK also act locally to regulate protein expression at activated synapses [17, 22]. Together, these findings provide new insight into the molecular mechanisms of epilepsy and explain how lack of pathway control results in seizures.

## RNA-binding proteins: mediators of remote expression control

Research in the last decade has shown that RNA-binding proteins (RBPs) are essential regulators of protein expression. For local gene expression, it was shown that RBPs regulate gene expression in a spatially restricted manner. mRNAs sequester RBPs by binding motifs in their 3′-untranslated region (3′-UTR). 3′-UTRs of localized transcripts are generally longer and more complex harboring cis-acting sequence motifs and secondary structures as binding platforms. It is commonly believed that some RBPs form large RNA–protein complexes termed RNA granules. These granules are transported along the cytoskeleton to synapses [23]. Upon stimulation, those particles are unpacked, the mRNA is released and locally translated. The process of remote expression control is mediated by RBPs. They control different steps of posttranscriptional regulation such as mRNA transport, translation and mRNA stability (reviewed in [24, 25]). The synchronization of all three steps is a prerequisite for local protein expression. Therefore, translation regulators such as Pumilio2 (Pum2) are part of transport granules as it was shown for Staufen2 (Stau2) [26]. The relevance of RBPs for the occurrence of neurological and neuropsychiatric diseases has risen in the last years. Both loss of function and gain of function mutations of RBPs result in epileptic phenotypes [27–29]. There is evidence that known epilepsy targets are under control of RBPs suggesting a local expression control of those mRNAs; probably at synapses. RBPs regulate mRNA distribution and protein synthesis of known epilepsy targets such as *CaMKIIα* or *KCNA1* encoding for the potassium channel Kv1.1, as well as multiple other ion channels [30]. It is therefore tempting to postulate that any aberrant translational control of newly synthesized proteins for regulators of synaptic excitability, e.g. ion channels or receptors, leads to epilepsy.

## Dysregulation of mTOR-mediated expression control leads to epileptic seizures

mTOR is a serine-threonine kinase that forms two protein complexes termed mTORC1 and mTORC2 [31]. mTORC1 consists of five components: mTOR as active center, raptor, MLST8, PRAS40 and DEPTOR. It is generally believed that mTOR acts mainly on translation. As a central check point, mTORC1 senses both internal and external signals such as nutrient and growth factor availability as well as oxidative stress to guide protein synthesis. mTORC2 is a rapamycin insensitive complex that contributes to cell survival functions, metabolism, proliferation and actin polymerization [31]. The exact role of mTORC2 in cellular signaling is still unclear. Several neuropathological diseases such as autism, depression and epilepsy have been linked to dysregulation of both complexes [17].

mTOR is a major player in the generation of neuronal homeostasis. Important factors for its maintenance is the balance of GABAergic and glutamatergic signaling, both of which are regulated by mTOR signaling [32]. Elevated activity of mTORC1 is implicated with increased neuronal excitability [31]. The relevance of mTOR regarding temporal lobe epilepsy in animal models and patients has been rising in importance throughout the last decade [33–35]. Various genetic diseases displaying a grave epileptic phenotype, like tuberous sclerosis, phosphatase and tensine homolog (PTEN)-hamartoma tumor syndromes and fragile X mental retardation syndrome (FXS), are associated with dysregulation of mTOR expression and activity [17]. Specifically, excessive mTOR signaling through a mutation in the tuberous sclerosis complex (TSC1/2) leads to hippocampal hyperexcitability linking mTOR with temporal lobe epilepsy [36]. Seizures generated in the hippocampus have also been related to hyperactive mTOR signaling in a mouse model harboring *PTEN* mutations. Knock-out of PTEN leads to hyperactive mTOR causing seizures generated in the hippocampus, mimicking the epileptic phenotype of focal cortical dysplasia [37]. Thus, controllability of excitability by mTOR is crucial to maintain balanced firing of neurons.

## Expression control by the MAPK pathway: a regulator of epileptic biomarkers

The mitogen-activated protein kinase (MAPK) family consists of three pathways, the extracellular signal regulated kinase (ERK) pathway, the p38 pathway and C-Jun N-terminal kinases (JNK) pathway. All three are highly conserved serine-threonine kinases that respond to nutrition and growth factors availability as well as neuronal activation [38]. ERK stimulates the expression of *N*-methyl-d-aspartate (NMDA) receptors causing synaptic excitability. This, in turn, leads to seizures [38]. Moreover, the neuroprotective effect of NMDA injected in mice before seizure induction is diminished by inhibition of the MAPK pathway, further underlining the potential of MAPK to regulate neuronal excitability [39]. Also the induction of mossy fiber sprouting, an effect causally associated with epileptogenesis, by the ERK/MAPK pathway has been shown in rats after traumatic brain injury [40]. This further supports a role of MAPK pathway in epileptogenesis. Interestingly, MAPK responds to different seizures-inducing treatments: maximal electroshock seizures in mice [41], kainate [42], pilocarpine [43] and pentylenetetrazole [44] as well as dopamine D1 receptor (D_1_R) agonist [45]. These findings suggest a role of MAPK in epileptogenesis.

Strikingly, there is evidence that also the MAPK pathway involves RBPs that are misregulated in epilepsy, like FMRP and HuR. In this context, kinases regulate those proteins and, therewith, their ability to control posttranscriptional gene expression [22, 46].

## mTOR and MAPK pathways control RBPs and thereby protein expression

**Fig. 1:**
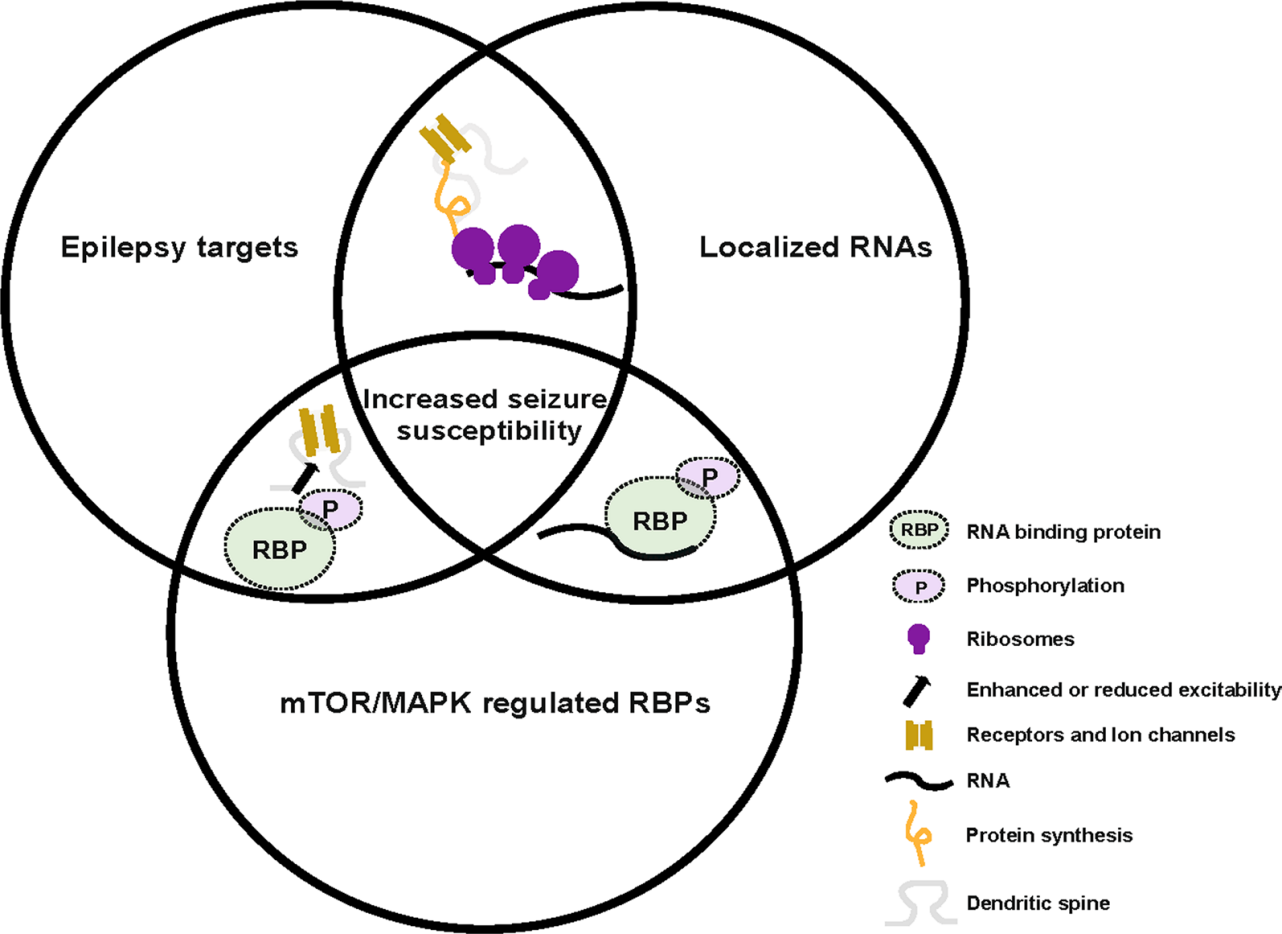
Venn diagram showing mTOR/MAPK mediated expression control via RBPs. Localized RNAs encoding for proteins involved in epilepsy are regulated by RBPs. Those, in turn, are guided by mTOR/MAPK

Some of the RBPs mentioned above are guided by mTOR/MAPK. Thus, it has been suggested that mTOR/MAPK affect local gene expression by regulating RBPs. In this review, we will focus on the effect of mTOR and MAPK on RBPs linking local expression control with neuronal hyperexcitability in epilepsy. We focus on localized transcripts at or near synapses that encode for proteins causing epilepsy. In addition, we emphasize that RBPs (1) regulate expression of those epilepsy targets and (2) are involved in the mTOR and/or MAPK pathway (Fig. 1). Together, mTOR/MAPK mediated control of RBPs represent a novel mechanism of remote expression control. In the following paragraphs, we will focus on RBPs already shown to be regulated by the mTOR and/or MAPK pathway.

## FMRP

Mutations in the fragile X mental retardation gene (*fmr1*) coding for the translation regulator FMRP lead to activity-dependent misregulation of protein synthesis at synapses and can result in seizures [27]. FMRP is an RBP involved in dendritic transport and translation control [47], specifically as a translational repressor [48]. It regulates the synthesis of calcium/calmodulin dependent protein kinase II alpha (CaMKIIα) that influences synaptic plasticity [49], the expression of multiple ion channels [50–54] and can lead causes epilepsy in knock-out mutant mice [55].

FMRP loss of function causes fragile X syndrome (FXS). Interestingly, FXS patients also suffer from seizures [56]. Furthermore, there is supportive evidence for a role of FMRP in temporal lobe epilepsy: In patients with temporal lobe epilepsy and in rats treated with the seizure-inducing drug pilocarpine, the level of cytosolic FMRP binding protein (CYFIP1) is elevated [57]. CYFIP is a protein expressed at synapses, displaying multiple functions like local regulation of actin cytoskeleton dynamics and dendritic spine morphology [57]. Interestingly, morphological abnormalities resulting from overexpressed CYFP1 can be rescued by the mTOR inhibitor rapamycin [58] linking mTOR activity with a direct FMRP interactor. Furthermore, it has been shown that elevated levels of phosphorylated mTOR is associated with decreased FMRP [59]. Additionally, regulation of FMRP via the mTOR target p70-S6 kinase (S6K1) has been proposed [60]. S6K1 phosphorylates FMRP and, thereby, it regulates its ability to repress translation. Surprisingly, the phosphorylation of FMRP has been reported to be mTOR independent [61] indicating an indirect effect of mTOR on FMRP-mediated expression control. Together, these results suggest that FMRP and mTOR are influencing each other to fine-tune protein expression.

The MAPK pathway is generally activated upon neuron stimulation. Activation of glutamate receptors, i.e. NMDA receptors, plays an important role in the generation of hyperactive firing of neurons. In this respect, activation of the metabotropic glutamate receptor 5 (mGluR5) results in excessive protein production in *fmr1* knockdown mice [20]. Interestingly, young FXS rats are susceptible to audiogenic seizures [62]. One hypothesis states that elevated protein levels at synapses leads to seizures [27, 63]. In contrast, mGluR5 does not directly influence chemically induced epilepsy in rats [64]. Inhibition of mGluR5 corrects symptoms of fragile X syndrome in adult mice and, importantly, reduces overactive ERK and mTOR signaling [65]. Furthermore, a direct link has been suggested from mGluR5 activation to mTOR and MAPK pathways in modulating FMRP-mediated expression control [66]. Importantly, the fragile X syndrome phenotype can be reversed by blocking ERK activity suggesting an interplay between FMRP and the ERK pathway [20, 62]. Since FXS-patient also suffer from seizures, those results indicate also a possible link to epilepsy.

## Hu protein family

Another group of RBPs connected to neurological disease is the Hu protein family (reviewed in [67]). Hu proteins are related to the embryonic lethal abnormal vision protein family (Elav) and were first described in patients with paraneoplastic encephalomyelitis [68]. These proteins are primarily responsible for neuronal differentiation, learning and memory, as well as long-term potentiation. They exert their RNA stabilizing function via three highly conserved RNA recognition motifs binding to AU-rich regions in the 3′-UTR, in the polyA tail [68] and in the coding region [69].

One member of the Hu family is HuD that regulates gene expression in neurons. HuD is upregulated after kainate-induced seizures in rats and shows activity dependent increase in protein levels in hippocampal neurons [70, 71]. Additionally, HuD localizes in an activity dependent manner in dendrites and interacts with mTOR targets in KCl stimulated hippocampal neurons [72]. DeBoer et al. showed that HuD knockout mice also show increased susceptibility to auditory seizures [28]. Supportive for these findings are results from other groups indicating a role of HuD in the regulation of synaptic excitability by adjusting the cellular glutamate levels via translation control of the glutaminase [73]. Unbalanced glutamate levels and related receptors are responsible for epileptogenesis [74]. Work by Sosanya et al. indicates an antagonistic relationship between HuD and mTOR in regulating expression of the voltage-gated potassium channel 1.1 (K_v_1.1) expression [69]. mTOR inhibition leads to degradation of HuD targets thereby increasing the concentration of free HuD molecules that are binding K_v_1.1 mRNA more efficiently. Hence, K_v_1.1 expression is increased upon mTOR-inhibition [21, 69]. Low Kv1.1 levels are associated with increasing seizure frequency in kainate induced temporal lobe epilepsy in rats, correlating with high mTOR levels [75].

HuD has been shown to control changes in synaptic plasticity via the local translation of synaptic proteins such as CaMKIIα [76]. Recent reports point to the interplay of the mTOR pathway with HuD-mediated expression control guiding branch specific expression of CaMKIIα [77]. Proper cellular and subcellular expression of CaMKIIα is crucial for neuronal excitability. Consequently, dysfunctions might contribute to epilepsy [78].

Importantly, inhibition of the protein kinase B (Akt)/glycogen synthase kinase 3 (GSK3) pathway leads to changes in HuD and HuR protein levels [76]. The GSK3 pathway therefore represents a link between the mTOR, Akt and MAPK pathways [79]. This suggests an interplay in the control of HuD between the different kinases. Together, these findings indicate that mTOR is necessary to guide HuD-mediated expression control. CaMKIIα, K_v_1.1 and glutaminase mRNAs are known HuD targets influencing neuronal excitability. Disruption of HuD expression control results in hyperactivity and seizures.

Additionally, neuronal activity causes HuD to localize in dendrites and dendritic spine like protrusions in primary hippocampal neurons [76]. Furthermore, it colocalizes with the eukaryotic translation initiation factor 4E (eIF4E) suggesting regulation of translation. Known as a target of mTOR mediated translation activation via 4E-binding protein (4EBP), elF4E is a point of convergence between the mTOR and the MAPK pathway. This was observed in mGluR dependent LTP. Activation of mGluR leads to ERK2 activation, which further phosphorylates and activates MAPK interacting kinase 2 (Mnk2) leading to eIF4E phosphorylation [80]. mGluR activation also recruits the phosphoinositide 3-kinase (PI3K)/mTOR pathway [81], releasing the 4E binding protein 2 (4EBP2) mediated inhibition of elF4E and promoting translation [80].

HuR is another well-studied member of the Hu family and ubiquitously expressed. Activation of the MAPK pathway by anisomycin causes stabilization of survival motor neuron (SMN) mRNA by HuR [82]. Furthermore, several studies reveal the MAPK pathway to regulate HuR [82–84]. In a mouse pentylenetetrazole model of epilepsy, HuR target mRNAs in the hippocampus were analyzed [85]. Amongst them were identified genes encoding for mossy fiber sprouting and apoptosis, which contribute to epilepsy [85]. In addition, *GAP*-*43* encoding for homonymous protein involved in axon growth is bound [86] and stabilized by HuR [76]. Importantly, GAP-43 is a marker for progressive epilepsy in patients with focal cortical dysplasia [87].

## CREB

cAMP response element binding protein (CREB) is a transcription activator [88]. It has been shown that it is crucial for the induction of LTP [89]. In a genome-wide microarray analysis of hippocampal tissue from patients with temporal lobe epilepsy, the protein kinase A (PKA)/CREB pathway appeared as one of the most enriched [90]. In addition, activation of CREB appears to cause epilepsy in rodent and human models [29]. Furthermore, CREB-regulated transcription coactivator 1 (CRTC1) has been shown to translocate into hippocampal nuclei following pilocarpine-induced status epilepticus [91]. CREB does not only influence transcription in the nucleus, but also leads to cell wide supply of protein and mRNA that can localize to the synapse when the synapse has been tagged, thereby permitting LTP [88, 89]. One transcript targeted by CREB is the brain derived neurotrophic factor (BDNF) [92], which has been associated to temporal lobe epilepsy in many cases [93] and may serve as a serum marker for epilepsy [94].

It is interesting to note that the MAPK pathway is a known regulator of CREB [40, 95]. Furthermore, the MAPK-CREB pathway induces mossy fiber reorganization after traumatic brain injury in rats, a characteristic feature of epileptogenesis [40]. In the human neocortex of epilepsy patients, a specific ERK activation pattern was linked to CREB phosphorylation and followed by enhanced transcription of CREB targets such as *BDNF* [96]. Additionally, increased synaptic density was observed in these layers linking synaptic morphology with CREB-induced *BDNF* transcription. Interestingly, it has been shown that BDNF induces synthesis of the early growth response factor 3 (EGR3) which regulates GABA receptors indicating a role of CREB in neuronal excitability [96]. These results highlight the causal link of MAPK activation and its effect on RBP-mediated expression control.

## Conclusions

**Fig. 2:**
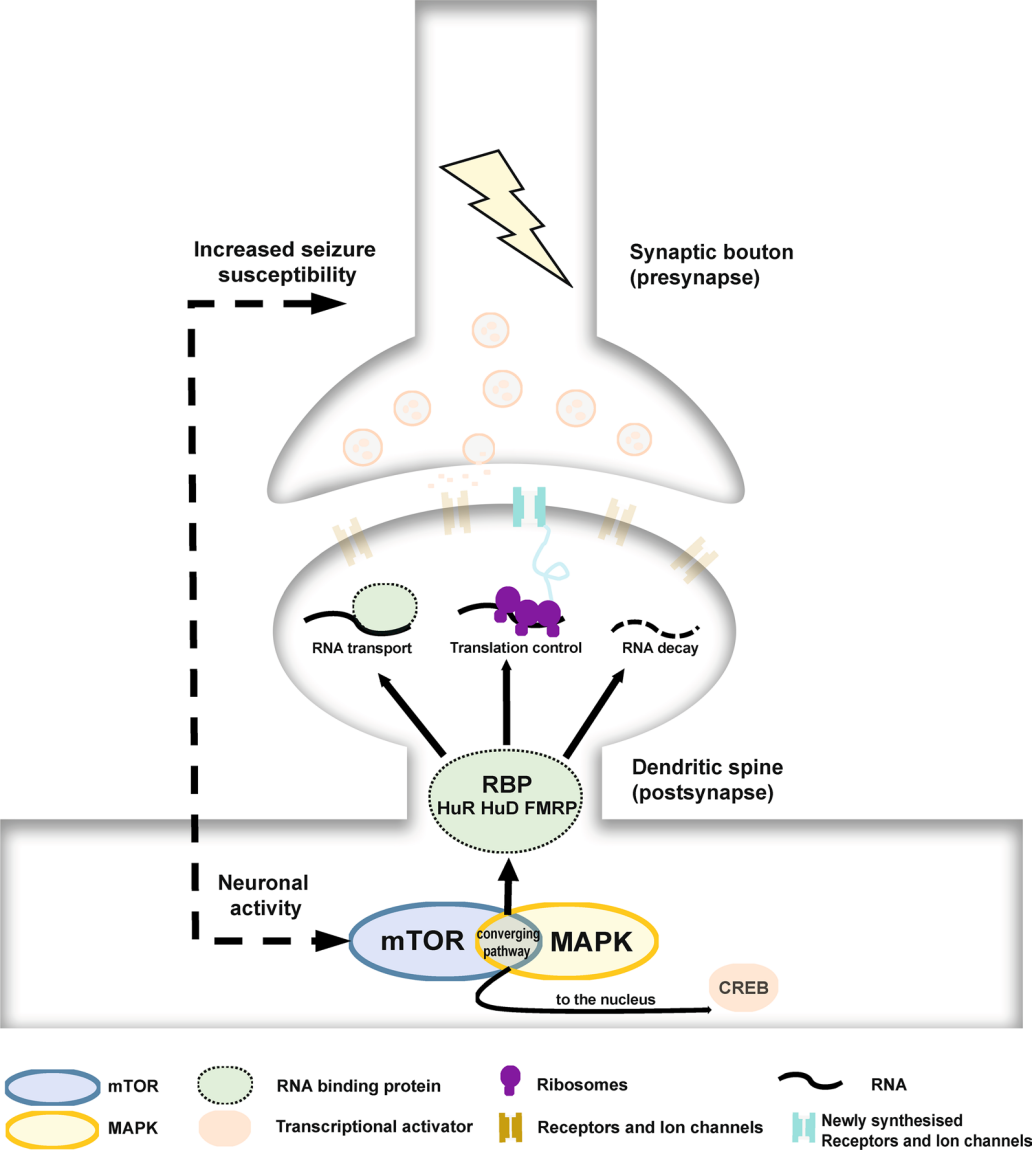
Model of mTOR and MAPK mediated RBP-dependent expression control. Regulation of spatially restricted protein expression is guided by RBPs. mTOR and MAPK both regulate RBPs. The kinases influence the ability of RBPs to control RNA stability and translation thereby affecting protein levels at the synapse. Misregulation of mTOR and MAPK pathways have been linked to epilepsy. We propose that disturbed regulation of local protein expression causes overburden levels of epilepsy factors resulting in hyperexcitability and eventually seizures

It is generally believed that epilepsy results from hyperexcitability and synchronous firing of neurons [7]. In the last decade, many biomarkers such as potassium and sodium channels as well as GABA receptors have been identified (Table 1). Beside those, mTOR and MAPK also contribute to epileptogenesis. Very often they are key players of converging pathways. Nonetheless, the underlying molecular mechanism of hyperexcitability and, in consequence, synchronous firing remain elusive. Strikingly, it has been shown that transcripts encoding for some of epileptic biomarkers including *mTOR* are localized in dendrites. Additionally, RBPs, mediators of RNA localization and remote expression control, have been linked to epilepsy bridging mTOR signaling with spatially restricted expression. We propose that hyperactive mTOR and MAPK affect RBPs and RBP-mediated expression control locally (Fig. 2). Thereby, they influence transcript transport, stability and translation at or near synapses of RBP target mRNAs. Thus, increased, local expression of ion channels and receptors results in enhanced synaptic density of excitability regulating proteins and, eventually, in increased synaptic transmission. In turn, augmented synaptic stimulation further activates mTOR and MAPK. This spatially restricted misregulation of synaptic excitability may therefore represent an accelerator of synchronous, neuronal firing that eventually results in seizures. Thus, RBPs might represent a novel category of epileptic biomarkers that can help to diagnose epilepsy in patients. Furthermore, they also can serve as additional targets for therapeutics. However, future studies will elucidate their eligibility for diagnosis and therapy.

In conclusion, remote expression control that is regulated by mTOR and MAPK provides new approaches to understand epilepsy at a molecular and cellular level. Here, mTOR possibly regulates RBPs, i.e. via direct phosphorylation, to guide their expression control abilities. This regulation of RBPs via mTOR may be affected by inhibiting drugs such as Rapamycin. A recent phase 3 study on Everolimus, a Rapamycin analog, shows a positive effect on therapy resistant focal epilepsy related to tuberous sclerosis [97]. This points to a significant role of mTOR inhibitors in epilepsy, and suggests a potential for the therapy of epilepsy caused by RBP deficiency. Nevertheless, more insight is necessary to develop new diagnosis and therapy strategies for the treatment of epilepsy and to understand side effects of mTOR and MAPK inhibition [98].

## Abbreviations

4EBP: 4E binding protein
Akt: protein kinase B
BDNF: brain derived neurotrophic factor
BK channel: calcium activated potassium channel
CaMKIIα: calcium calmodulin dependent protein kinase II alpha
Cav2.2: voltage gated calcium channel 2.2
CREB: cAMP response element binding protein
CRTC1: CREB-regulated transcription coactivator 1
CYFIP1: cytosolic FMRP binding protein
EGR3: early growth response factor 3
eIF4E: eukaryotic translation initiation factor 4E
Elav: embryonic lethal abnormal vision protein family
ERK: extracellular signal regulated kinase
Fmr1: fragile X mental retardation gene
FXS: fragile X mental retardation syndrome
GABA: gamma-aminobutyric acid
GAP-43: growth associated protein 43
GSK3: glycogen synthase kinase 3
JNK: C-Jun-N-terminal kinase
Kv1.1: voltage-gated potassium channel 1.1
Kv3.1: voltage-gated potassium channel 3.1
Kv4.2: voltage-gated potassium channel 4.2
MAPK: mitogen-activated protein kinase
mTOR: mechanistic target of rapamycin
mGluR: metabotropic Glutamate receptor
NMDA: *N*-methyl-d-aspartate
PI3K: phosphoinositide 3-kinase
PKA: protein kinase A
PTEN: phosphatase and tensine homolog
RBP: RNA binding protein
S6K: p70-S6 kinase
Slack channel: Slack sodium activated potassium channel
SMN: survival motor neuron
TLE: temporal lobe epilepsy
TSC: tuberous sclerosis complex
